# Personalized Genome‐Scale Modeling Reveals Metabolic Perturbations in Fibroblasts of Methylmalonic Aciduria Patients

**DOI:** 10.1002/jimd.70077

**Published:** 2025-08-11

**Authors:** Almut Heinken, Hussein Awada, Vito R. T. Zanotelli, D. Sean Froese, Rosa‐Maria Guéant‐Rodriguez, Jean‐Louis Guéant

**Affiliations:** ^1^ UMRS Inserm 1256 nGERE (Nutrition‐Genetics‐Environmental Risks), Institute of Medical Research (Pôle BMS) University of Lorraine Nancy France; ^2^ Division of Metabolism and Children's Research Center, University Children's Hospital Zürich University of Zürich Zürich Switzerland; ^3^ National Reference Medical Biology Laboratory (LBMR) for Inherited Metabolic Diseases, Biochemistry‐Molecular Biology‐Nutrition Laboratory University Regional Hospital of Nancy Nancy France; ^4^ Department of Hepato‐Gastroenterology, National Reference Center for Inherited Metabolic Diseases University Regional Hospital of Nancy Nancy France; ^5^ Joan Klein Jacobs Center for Precision Nutrition and Health Cornell University Ithaca New York USA

## Abstract

Cobalamin (vitamin B12) is an essential cofactor for two human enzymes, methionine synthase and methylmalonyl‐CoA mutase. Inborn errors of cobalamin metabolism (IECMs) are inherited genetic defects resulting in improper transport, modification, or utilization of cobalamin and include inherited methylmalonic acidurias, a group of IECMs most frequently caused by a defect in the methylmalonyl‐CoA mutase enzyme. Here, we performed genome‐scale modeling of IECMs to gain insight into their metabolic perturbations. First, we simulated deficiencies in 11 IECM‐related genes and demonstrated that they cluster based on impaired metabolic pathways. Next, we leveraged RNA sequencing data from fibroblasts of 202 individuals with methylmalonic aciduria and 19 unaffected controls to construct and interrogate personalized metabolic models. Finally, we analyzed fluxes differing between patients depending on reported symptom presentation. Our findings reveal that (i) metabolic pathways including fatty acid metabolism and heme biosynthesis have reduced flux in IECMs, (ii) in personalized simulations, succinate and fumarate production and heme biosynthesis are impaired, especially in methylmalonyl‐CoA mutase deficiency, (iii) one‐carbon metabolism reactions such as serine hydroxymethyltransferase and folylglutamate synthase have reduced flux in all individuals with methylmalonic aciduria, and (iv) specific metabolic pathways are up‐ or down‐regulated according to symptoms, including failure to thrive and hematological abnormalities, and treatments, such as antibiotics and protein restriction. Overall, our study delineates metabolic pathways perturbed in IECMs. In future applications, our modeling framework could be applied to other rare genetic diseases or used to predict personalized therapeutic or dietary interventions.

## 1 Introduction

Vitamin B12 (cobalamin) is a cofactor with a complex structure containing a central cobalt atom [1]. It is essential in man and needs to be taken up through the diet from foods of animal origin [2]. Cobalamin is bioavailable in the diet in the form of methylcobalamin or hydroxocobalamin and is present in the form of cyanocobalamin pharmaceutically [2]. Hydroxocobalamin and cyanocobalamin are converted into adenosylcobalamin, the active cofactor for methylmalonyl‐CoA mutase in the mitochondrion (EC 5.4.99.2, encoded by *MMUT*) and methylcobalamin, the active cofactor for methionine synthase in the cytosol (EC 2.1.1.13, encoded by *MTR*) [2]. Methylmalonyl‐CoA mutase catalyzes the conversion of L‐methylmalonyl‐CoA to succinyl‐CoA, which is a key step in the propionate anaplerotic pathway of the tricarboxylic acid (TCA) cycle [2]. Methionine synthase catalyzes the remethylation of homocysteine to methionine using 5‐methyltetrahydrofolate as a methyl donor [1]. Besides its role as a proteogenic amino acid, methionine serves as the substrate for methionine adenosyltransferase (EC 2.5.1.6), which synthesizes the universal methyl donor *S*‐adenosylmethionine (SAM) [1]. Methionine synthase also links the methionine cycle with the folate cycle [1]. Taken together, cobalamin plays a key role in central metabolic pathways such as one‐carbon metabolism, propionate metabolism, and the TCA cycle, and along with folate, is essential for the epigenetic machinery [1, 2].

Inherited defects of cobalamin metabolism (IECMs) constitute a subgroup of inborn errors of metabolism (IEMs) with 19 known diseases and are caused by defects in genes involved in cobalamin absorption, transport, recruitment, and regeneration [3, 4]. IECMs are associated with a variety of severe symptoms, including neurological, hematological, and metabolic, with great variability between patients and disease types [4]. For instance, a recent study found that patient fibroblasts from cblC, epiCblC, and CblG subtypes of IECMs had distinct metabolic profiles in a multi‐omics analysis [5]. IECMs related to the mitochondrial role of cobalamin typically result in the phenotype of isolated methylmalonic aciduria (MMA) associated with elevated levels of methylmalonic acid in blood and urine [6]. This includes complete or partial defects in the gene encoding the methylmalonyl‐CoA mutase enzyme (mut^0^ and mut^−^ types) [6]. Recently, a multi‐omics analysis of fibroblasts from 210 MMA patients and 20 healthy controls revealed altered metabolic pathways and metabolite concentrations in patients [7].

IEMs have long been considered simple, with little interindividual variation, according to the one gene‐one disease paradigm [8]. More recently, it has been recognized that, not unlike in multifactorial diseases, environmental factors including diet, lifestyle, and the gut microbiome can influence the severity of symptoms in IEMs [8]. Precision medicine, or medicine that takes individual factors such as genetics, diet, exposures, medication, and the microbiome into account, has been proposed as a new paradigm for medicine [9]. A key feature of precision medicine is the stratification of patients based on comprehensive characterization through omics data, for example, transcriptomics, proteomics, and metabolomics [9, 10]. While initially conceived for common multifactorial diseases, precision medicine has also been proposed to be valuable for the stratification of patients affected by inherited rare diseases [10]. To integrate multi‐omics data into predictive models that can propose testable hypotheses for IEM pathogenesis and treatment, systems biology methods are needed [8].

Constraint‐based Reconstruction and Analysis is a bottom‐up systems biology approach that utilizes detailed genome‐scale reconstructions to predict a space of feasible fluxes [11]. These genome‐scale reconstructions contain biochemical reaction networks based on genetic, biochemical, and physiological knowledge of the target organism, and take the form of a stoichiometric matrix [11]. Through the application of physicochemical, thermodynamic, environmental, and condition‐specific constraints including multi‐omics data, genome‐scale reconstructions can be converted into mathematical models [11]. One such genome‐scale reconstruction, deemed Recon3D, is a comprehensive computational representation of the metabolic enzymatic and transport reactions taking place in any human cell, accounting for 3288 open reading frames and 13 543 metabolic reactions involving 4140 unique metabolites [12]. Recon3D was built through extensive manual curation spanning over 10 years of development with multiple precursors [12]. Through appropriate parameterization, human genome‐scale reconstructions such as Recon3D enable building tissue‐specific models as well as context‐specific models representing disease and control states [13]. Specifically, the loss of function through gene defects, such as in IEMs, can be simulated by preventing flux through reactions associated with the respective gene [13]. This has enabled simulating the outcomes of metabolic blocks caused by gene defects in their metabolic context for a variety of IEMs (reviewed recently in [14]). For instance, a recent study by Ramon et al. used the human genome‐scale reconstruction to simulate a complete defect in the *MMUT* gene [15]. The model predicted decreased catabolism of branched‐chain amino acids, but increased anapleurotic fluxes into the TCA cycle for the *MMUT* knockout [15].

Besides environmental constraints such as nutrient availability, genome‐scale models can be further tailored to be condition‐specific through the integration of omics data, e.g., transcriptomics, proteomics, or metabolomics [16]. Omics data integration effectively results in the reduction of the feasible flux space by pruning a context‐specific subnetwork from a genome‐scale model [16]. Such generation and interrogation of patient‐specific models through integration of omics data have been applied to a variety of complex diseases, e.g., cancer [17] or nonalcoholic fatty liver disease [18]. Yet, to our knowledge, all previous applications of genome‐scale modeling to simulating IEMs considered patients' metabolic fluxes as defined entirely by the causal gene with no inter‐individual variation [14].

Previously, we have proposed personalized metabolic modeling through omics data integration as a useful tool for the elucidation of patient‐specific metabolic phenotypes in rare diseases, analogous to the established procedures for common diseases [14]. Here, we used genome‐scale modeling to elucidate the metabolic disturbances associated with IECMs. First, we expanded the global human genome‐scale reconstruction, Recon3D, and a tissue‐specific reconstruction of the human fibroblast with cobalamin absorption, transport, conversion, and cofactor utilization reactions. Next, we predicted the impaired metabolic fluxes associated with 11 known IECMs as well as cobalamin deficiency. Finally, we retrieved RNA sequencing data from fibroblasts of 202 individuals with MMA and 19 unaffected controls. We constructed personalized models for each sample by integrating RNA sequencing data into the tissue‐specific genome‐scale reconstruction. We then predicted the metabolic fluxes for each personalized model and performed statistical analyses to identify pathways differing between patients and controls. Our results demonstrated that a wide variety of pathways beyond those directly related to the affected reaction were perturbed. Moreover, we leveraged available metadata for patients to identify differential fluxes depending on the presence or absence of symptoms. Taken together, we present a comprehensive, disease‐ and patient‐specific view of metabolic pathways altered in IECMs. To our knowledge, this is the first study to elucidate patient‐specific disturbed metabolic fluxes in a rare disease through constraint‐based modeling.

## 2 Methods

### 2.1 Simulations

All performed reconstruction refinement and simulations were carried out using functions implemented in the COBRA Toolbox [19], using MATLAB (Mathworks Inc.) version 2020b and IBM CPLEX (IBM Inc.) as the linear programming solver.

### 2.2 Formulation of Cobalamin Metabolism Pathway

To identify cobalamin‐dependent reactions currently absent in Recon3D, existing reactions containing cobalamin were inspected using the Virtual Metabolic Human (VMH) website [20]. Additional metabolites and reactions for cobalamin absorption, transport, conversion, recruitment, and release were formulated based on the available literature [1, 2, 21]. Mass and charge balance was ensured by using the rBioNet tool [22]. In total, 10 metabolites, 24 reactions, and seven genes were added to Recon3D [12] and to a fibroblast‐specific genome‐scale reconstruction derived from Recon3D [23]. The added reactions, metabolites, and genes are shown in Table S1.

### 2.3 Simulation of IECM Phenotypes

The flux consistent Recon3D model supplemented with cobalamin metabolism was used to predict altered fluxes in IECMs. After expansion, Recon3D accounted for 11 genes corresponding to known IECMs (Table 1). Each IECM was modeled by simulating a complete knockout of the corresponding gene in Recon3D through the *deleteModelGenes* function in the COBRA Toolbox. Cobalamin deficiency was simulated by setting the lower bounds for all exchange reactions of cobalamin metabolites to zero. Flux variability analysis (FVA) [24] was performed to retrieve the flux spans for all reactions. The biomass objective function was used as the objective with 99% optimal fluxes required. Reactions with an altered minimal or maximal flux of at least 10% compared with the wild type were considered to be affected by the knockout or deficiency.

**TABLE 1:** List of known IECMs captured in the revised Recon 3D and fibroblast genome‐scale reconstruction.

Complementation group	Gene name	Associated IECM	OMIM ID
cblA	*MMAA*	Methylmalonic aciduria type cblA	251100
cblB	*MMAB*	Methylmalonic aciduria type cblB	251110
cblC	*MMACHC*	Methylmalonic aciduria and homocystinuria type cblC	277400
cblD	*MMADHC*	Methylmalonic aciduria and homocystinuria type cblD	277410
cblE	*MTRR*	Homocystinuria‐megaloblastic anemia type cblE	236270
cblF	*LMBRD1*	Methylmalonic aciduria and homocystinuria type cblF	277380
cblG	*MTR*	Homocystinuria‐megaloblastic anemia type cblG	250940
cblJ	*ABCD4*	Methylmalonic aciduria and homocystinuria type cblJ	614857
CD320	*CD320*	Methylmalonic aciduria due to transcobalamin receptor defect	613646
mut	*MMUT*	Methylmalonic aciduria due to methylmalonyl‐CoA mutase deficiency	251000
TCN2	*TCN2*	Transcobalamin II deficiency	275350

*Note:* The table shows the complementation group name, gene name, associated disease name, and Online Mendelian Inheritance in Man (OMIM) ID.

Abbreviation: IECM = inborn error of cobalamin metabolism.

### 2.4 Construction of Personalized Models

A fibroblast‐specific human reconstruction [23] supplemented with the cobalamin pathway (see above) was used to build patient‐specific models. RNA sequencing data from fibroblasts of 202 MMA patients and 19 healthy controls was retrieved [7]. The data was converted from RPKM format was converted into TPM format. Next, gene symbols in the RNA sequencing data were mapped onto the gene locus IDs contained in the fibroblast reconstruction. Genes that were not present in the fibroblast reconstruction were removed. Subsequently, personalized models were generated using rFASTCORMICS [25]. Briefly, rFASTCORMICS uses gene expression levels from RNA sequencing data to define a set of active reactions. Subsequently, a context‐specific model is extracted and gap‐filled to enable flux through the biomass objective function. rFASTCORMICS was run using standard input parameters, following the workflow described at https://github.com/sysbiolux/rFASTCORMICS. A list of metabolites contained in Dulbecco's modified Eagle's medium (DMEM), which fibroblast cultures had been cultivated in [7], was used to define medium components for rFASTCORMICS. In total, 221 personalized models were generated. To further personalize the models, RNA sequencing data was used to adjust reaction constraints using eFlux [26]. Briefly, eFlux enforces that the allowed minimal and maximal fluxes are proportional to the relative gene expression. The eFlux implementation in the COBRA Toolbox was used in a modified version in which maximal lower and upper bounds of −1000 and 1000, respectively, were allowed.

### 2.5 Interrogation of Personalized Models

For each personalized model, minimal and maximal fluxes for each reaction were predicted through FVA as described above. Growth on DMEM medium was simulated by assigning the appropriate constraints (Table S2) on the corresponding exchange reactions and setting the lower bounds on all other exchange reactions to zero. All reactions that had nonzero minimal or maximal flux in at least one personalized model were retained for further analyses. To determine the theoretical maximal production potential for metabolites, a sink reaction was added separately for each nonunique metabolite, set as the objective function, and flux balance analysis was performed while simulating DMEM medium.

### 2.6 Statistical Analysis

Disease diagnoses and clinical features for each patient were available [7]. A Wilcoxon rank sum test was performed in MATLAB using the ranksum function to determine metabolic fluxes differing between groups. Correction for multiple testing was performed using the mafdr function in MATLAB after excluding reactions or metabolites with zero or constant flux across all compared samples. Comparisons included (i) MMA patients and healthy controls, (ii) mut‐type MMA patients and other types of MMA, (iii) mut^0^ and mut^−^ cases, and (iv) presence and absence of reported clinical symptoms in mut‐type MMA patients and other types of MMA. For (i), all MMA patients and healthy controls were considered. For (ii), mut‐type MMA patients and other types of MMA patients were considered. For (iii), only mut‐type MMA cases were considered, and mut^0^ and mut^−^ were compared. For (iv) mut‐type MMA patients and other types of MMA patients were compared separately for the presence and absence of reported symptoms. Only symptoms present in at least eight patients in the respective group were considered. Note that absence of symptoms may indicate either absent or not reported in patient documentation. Moreover, the available information on symptoms or treatments was not used to modify the models in any way.

### 2.7 Visualization

Plots were generated in MATLAB version R2020b, R version 4.3.0, BioRender, and https://bioinformatics.psb.ugent.be/webtools/Venn/.

## 3 Results

### 3.1 Refinement of the Cobalamin Absorption and Utilization Pathway in the Human Genome‐Scale Reconstruction

**FIGURE 1:**
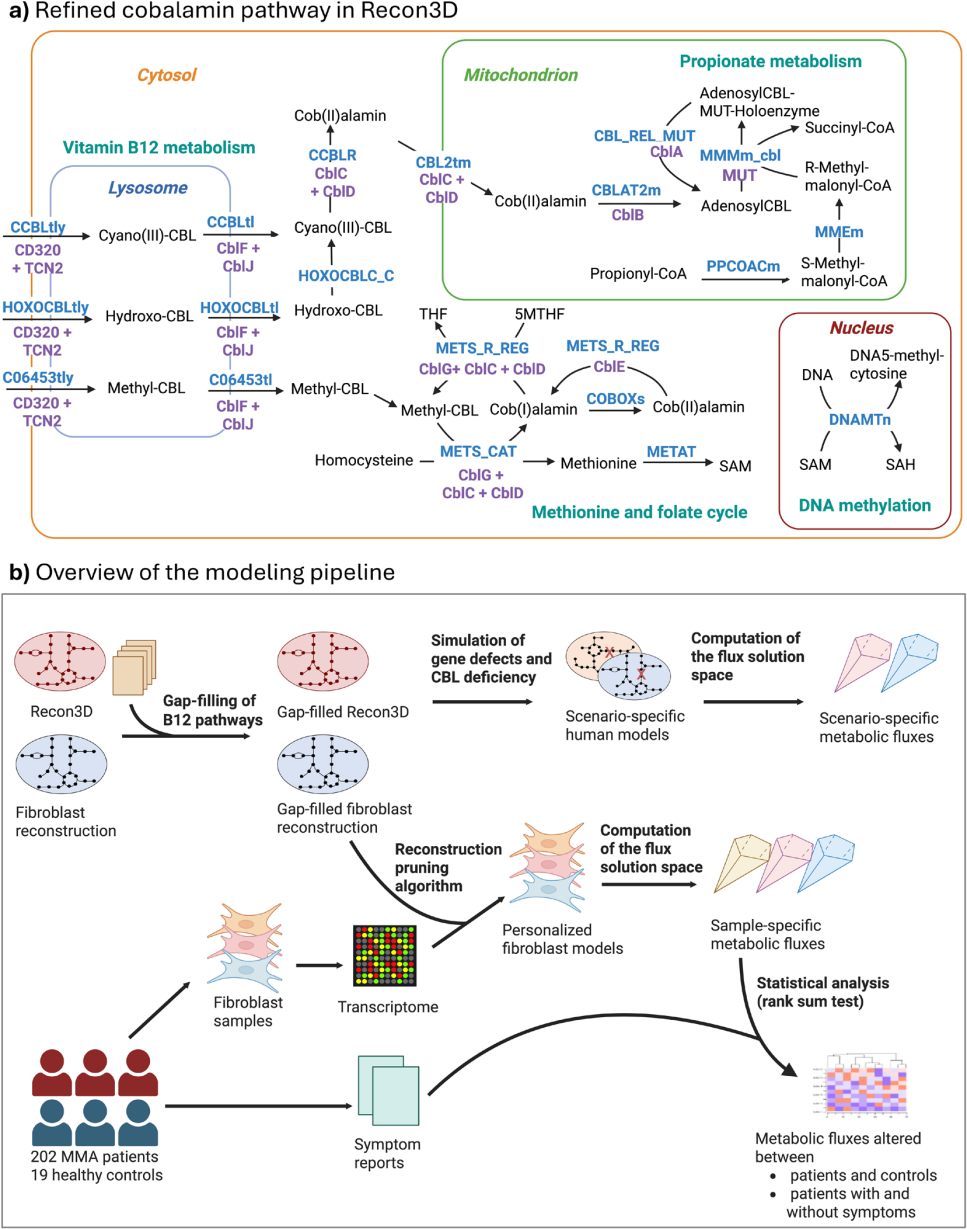
Overview of the performed model refinement and simulations. (a) Overview of the refined vitamin B12 absorption and transport, methionine cycle, and folate cycle pathway in Recon3D and the fibroblast reconstruction. Genes associated with known IECMs that were simulated in silico are shown in purple. Reactions are shown in blue by VMH reaction ID [20] or reaction ID newly assigned in this study (Table S1). (b) Consequences of IECMs on metabolic pathways. 1CM = one‐carbon metabolism, CBL = cobalamin, SAH = *S*‐adenosylhomocysteine, SAM = S‐adenosylmethionine, THF = tetrahydrofolate. (b) Overview of the modeling and analysis pipeline. Created in https://BioRender.com.

The reaction stoichiometry of cofactor utilization in the human genome‐scale reconstruction Recon3D [12] is partially incomplete, limiting its potential for modeling IEMs of vitamin and cofactor metabolism, as previously shown for flavin‐dependent reactions [27]. Indeed, upon inspection, the reaction stoichiometry for methionine synthase (VMH ID: METS) and methylmalonyl‐CoA mutase (VMH ID: MMMm) in Recon3D did not explicitly account for cobalamin as a cofactor. Moreover, cobalamin absorption, transport, and conversion reactions were incomplete. To explicitly account for the role of cobalamin in human metabolism, the cobalamin pathway in Recon3D was expanded (see Section 2). Hence, even extensively manually curated genome‐scale reconstructions such as Recon3D still contain metabolic gaps that can be closed through further refinement. The refined pathway is shown schematically in Figure 1a. We then devised a workflow to systematically evaluate the consequences of IECMs on metabolic pathways (Figure 1b).

### 3.2 In Silico Gene Defect Phenotypes Reflect IECM Subtypes

**FIGURE 2:**
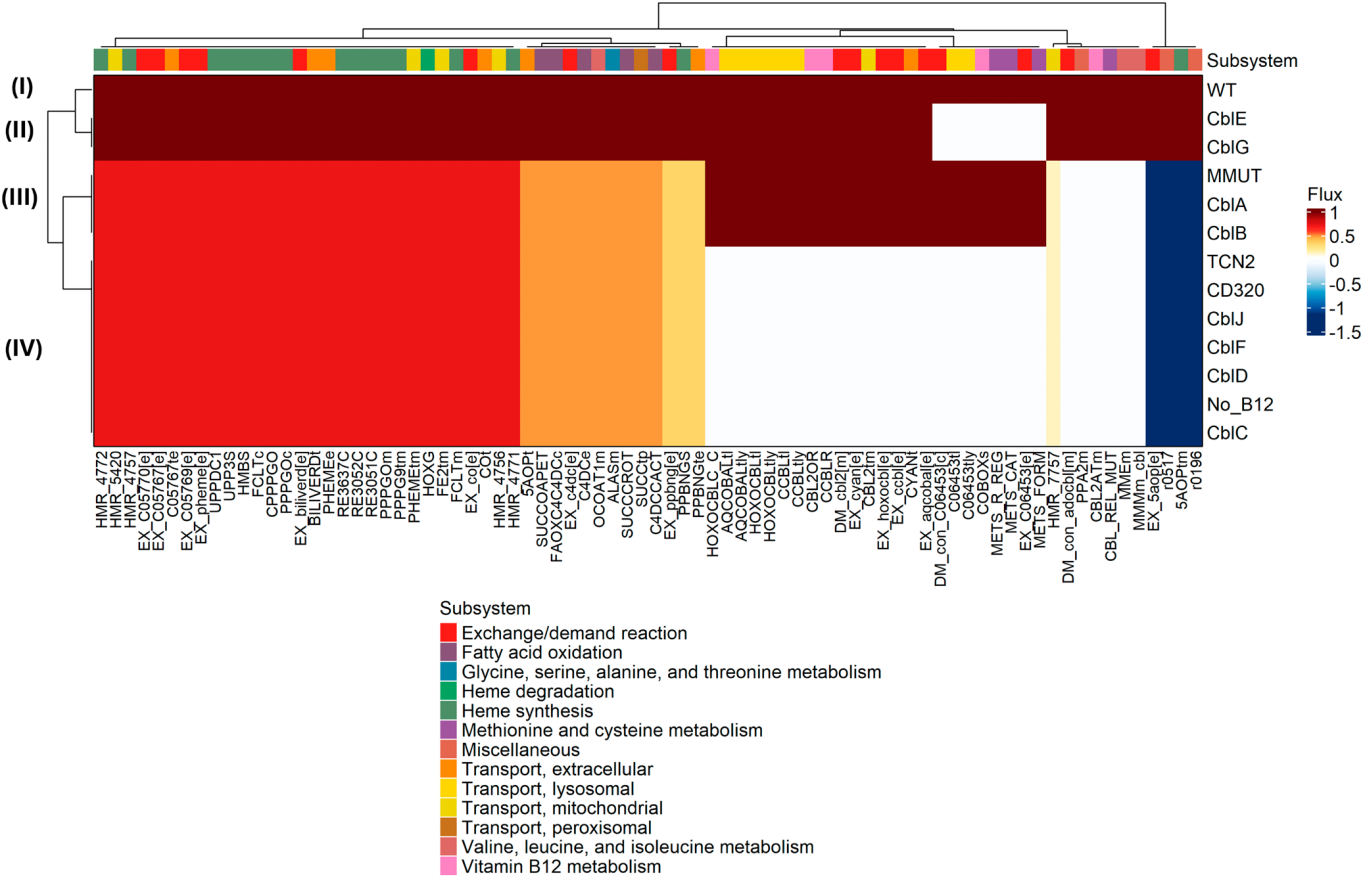
Altered fluxes in silico in the modeled scenarios associated with IECMs. Shown are reactions that were reduced in flux in at least one scenario by at least 5%. Fluxes are shown as a fraction of flux seen in the wild type. The four clusters corresponding to symptom groups are indicated as follows: (I) WT, (II) “Remethylation” subtype, (III) “Mitochondrion” subtype, (IV) “B12 bioavailability” and “Cytoplasmatic transport” subtypes. No_B12 = cobalamin deficiency, WT = wild type. Reactions are displayed by VMH reaction ID [20] or reaction ID newly assigned in this study (Table S1).

The phenotype of enzymopathies in a hypothetical patient can be evaluated through metabolic modeling by simulating a knockout of the relevant gene [14]. Of the 14 gene defects that have been associated with disease outcomes related to cobalamin [21], 11 are captured by the expanded fibroblast reconstruction (Table 1). We simulated the effect of a complete defect in each gene for fibroblasts and predicted the flux spans (minimal and maximal fluxes) for each reaction in each gene defect model (see Section 2). Flux spans were also predicted for the wild type (WT) situation, in which no gene was deleted, and for a model of cobalamin deficiency in which uptake was prevented (see Section 2). Predicted flux ranges in each scenario were then compared, and fluxes that differed by at least 5% from wild type in at least one scenario were identified (81 reactions total, Figure 2, Table S3). The fluxes affected in the modeled scenarios formed four clusters (I–IV, Figure 2).

Recently, Wiedemann et al. grouped IECMs into four clusters based on symptom reports, namely: B12 availability, cytoplasmic transport, remethylation, and the mitochondrion, each of which varied in disease presentation [4]. We compared the clusters predicted by the modeling (Figure 2) with the symptom‐based clustering of IECMs into multiple subtypes by Wiedemann et al. [4] and found three overlapping in silico clusters. Cluster (I) consisted only of the WT. Cluster (II) contained methionine synthase and methionine synthase reductase, which form the “Remethylation” subtype [4]. Cluster (III) contained knockouts related to methylmalonyl‐CoA mutase and corresponded to the “Mitochondrion” subtype of IECM phenotypes [4]. Finally, Cluster (IV) contained genes involved in cobalamin absorption, transport, and conversion, as well as deficiency; hence, corresponding to the “B12 bioavailability” and “Cytoplasmatic transport” subtypes [4]. As expected, knockouts in Cluster (IV) had the broadest effect on fluxes (Figure 2). Reactions belonging to the subsystems of cobalamin transport, metabolism, and methionine and cysteine metabolism (eight, four, and three reactions, respectively) could not carry flux in Cluster (IV) but were not affected in Cluster (III) (Figure 2). Hence, the simulations reflected the essential role of cobalamin availability for the methionine cycle [1]. The two half‐reactions of methionine synthase (METS_FORM, METS_CAT) as well as methionine synthase reductase (METS_R_REG), as expected, did not carry flux in Clusters (II) and (IV). Fluxes in methylmalonyl‐CoA mutase (MMMm_cbl) and methylmalonyl‐CoA epimerase (VMH ID: MMEm) were only affected in Clusters (III) and (IV) (Figure 2). Moreover, heme degradation and heme synthesis reactions were reduced in flux in Clusters (III) and (IV). Succinyl‐CoA is the precursor for heme synthesis via the 5‐aminolevulinate synthase (VMH ID: ALASm). Consequently, reduced succinyl‐CoA availability due to impaired methylmalonyl‐CoA mutase activity also caused a reduction in the final step for heme biosynthesis (VMH ID: FCLTm) (Figure 2). Heme is the main component of hemoglobin; consistently, hematological symptoms such as anemia are common in IECM patients, particularly in the cobalamin bioavailability cluster [4]. Finally, reactions in fatty acid metabolism that can use succinyl‐CoA as a substrate were also impaired in flux in Clusters III and IV, namely, carnitine O‐octanoyltransferase (VMH ID: SUCCCROT), carnitine palmitoyltransferase (VMH ID: FAOXC4C4DCc), 3‐oxoacid CoA transferase (VMH ID: OCOAT1m), and thioesterase (VMH ID: SUCCOAPET) (Figure 2). To summarize, a variety of metabolic pathways, including fatty acid metabolism and heme biosynthesis, were affected by impaired flux through the cobalamin absorption, transport, and utilization pathway. Fluxes altered in IECMs reflected the classification into subtypes based on symptoms that had recently been defined [4].

### 3.3 Personalized Modeling of IECM Patients

While in silico gene deletions are a useful tool for predicting fluxes affected by enzymopathies, they cannot capture every aspect of metabolic changes in patients. For one, they only capture reduced fluxes in reactions directly related to the defective protein according to reaction stoichiometry. Reaction fluxes altered due to, for example, impaired epigenetic regulation of pathways, as can be expected in IECMs due to their link to one‐carbon metabolism, will not be accounted for. Second, individual‐specific differences in patients, such as milder or more severe metabolic perturbations, will not be captured. To overcome these limitations, we leveraged RNA sequencing data from fibroblasts of 202 individuals affected by MMA and 19 unaffected controls [7]. Of the 202 affected individuals, 143 presented with a partial or complete deficiency in methylmalonyl‐CoA mutase activity [7] (deemed “mut^−^” and “mut^0^,” respectively, in the following sections). The remaining 59 (deemed “MMA_Other” in the following sections) had other causes of MMA, namely, acyl‐CoA synthetase deficiency (20 cases), succinyl‐CoA ligase deficiency (three cases), transcobalamin 2 (TCN2) deficiency (three cases), cblA‐type MMA (two cases), cblB‐type MMA (one case), and unspecified MMA (30 cases) [7]. We constructed personalized models for the 221 samples by integrating the gene expression levels that had been determined through RNA sequencing into a tissue‐specific genome‐scale reconstruction of the human fibroblast [23] (see Section 2). On average, the resulting personalized genome‐scale models contained 3077.87 reactions, 2246.55 nonunique metabolites, and 1519.57 genes (Table S4). Model sizes were comparable between controls and MMA, and no statistically significant differences between groups were found.

Subsequently, we predicted flux spans (minimal and maximal fluxes) for all reactions present in each personalized model (see Section 2). Note that by convention, positive flux corresponds to flux in the forward direction, and negative flux corresponds to flux in the reverse direction. Finally, we predicted the theoretical potential of each personalized model to synthesize each nonunique metabolite present in the model. In total, 3922 reactions were present in at least one personalized model, of which 1038 showed nonzero minimal flux, and 3684 showed nonzero maximal flux in at least one model in simulations (Table S5).

### 3.4 Reactions With Impaired Flux in Subtypes of Patients

To identify disturbed metabolic fluxes in MMA, we performed a Wilcoxon rank sum test followed by correction for false discovery rate on minimal and maximal reaction fluxes for MMA subtypes (mut^0^, mut^−^, and MMA_Other) and controls (see Section 2). In total, 53 minimal or maximal fluxes differed significantly between mut^0^ and controls after correction for multiple testing (*p* value adjusted for false discovery rate < 0.05); all of which had higher flux in controls (Table 2, Table S6). Compared with controls, mut^0^ derived fibroblasts had reduced flux in multiple subsystems including heme synthesis, transport, folate metabolism, and valine, leucine, and isoleucine metabolism (Table 2). Between controls and mut^−^, 397 minimal or maximal reaction fluxes remained significant after correction for multiple testing (Table 2, Table S7). Between mut^0^ and mut^−^ subtypes, six reactions remained significant after correction for multiple testing, all of which, as expected, had higher flux in mut^−^ (Table 2, Table S8). Between controls and non‐mut‐type MMA cases, 90 reaction fluxes remained significant after correction for multiple testing (Table 2, Table S9). Interestingly, there was only a partial overlap between the 53 reactions significantly affected in mut^0^ patient fibroblast models and the 51 reactions reduced in flux in the simulated theoretical mut^0^ knockout shown in Figure 2 (27 reactions overlapping, Figure S1).

**TABLE 2:** Reactions that differed significantly after correction for multiple testing between compared groups of samples.

Subsystem	Higher in controls vs. mut0	Higher in controls vs. mut^−^	Higher in mut^−^ vs. controls	Higher in controls vs. other MMA	Higher in mut^−^ vs. mut0
Butanoate metabolism	1	0	0	0	0
Cholesterol metabolism	2	61	1	0	0
Citric acid cycle	1	1	0	0	0
Eicosanoid metabolism	0	1	0	0	0
Exchange/demand reaction	4	6	1	1	1
Fatty acid oxidation	0	137	0	41	0
Fatty acid synthesis	0	57	0	20	0
Folate metabolism	6	8	0	6	0
Glycerophospholipid metabolism	1	1	1	0	0
Glycine, serine, alanine, and threonine metabolism	2	3	0	0	0
Glycolysis/gluconeogenesis	0	1	0	1	0
Heme synthesis	14	14	0	0	0
Methionine and cysteine metabolism	1	1	0	0	1
Miscellaneous	2	3	0	1	1
Nucleotide interconversion	0	1	0	0	0
Pentose phosphate pathway	0	1	0	0	0
Pyrimidine synthesis	0	1	0	0	0
Squalene and cholesterol synthesis	0	1	0	0	0
Transport	13	70	4	20	0
Tryptophan metabolism	3	0	0	0	0
Valine, leucine, and isoleucine metabolism	2	3	18	0	2
Vitamin B12 metabolism	1	1	0	0	1

*Note:* Reactions are grouped by assigned metabolic subsystem in the Recon3D genome‐scale reconstruction. More details are shown in Tables S5–S9.

### 3.5 Heme Biosynthesis, Branched‐Chain Amino Acid Metabolism, Fatty Acid Metabolism, and One‐Carbon Metabolism Are Altered in Flux in MMA

**FIGURE 3:**
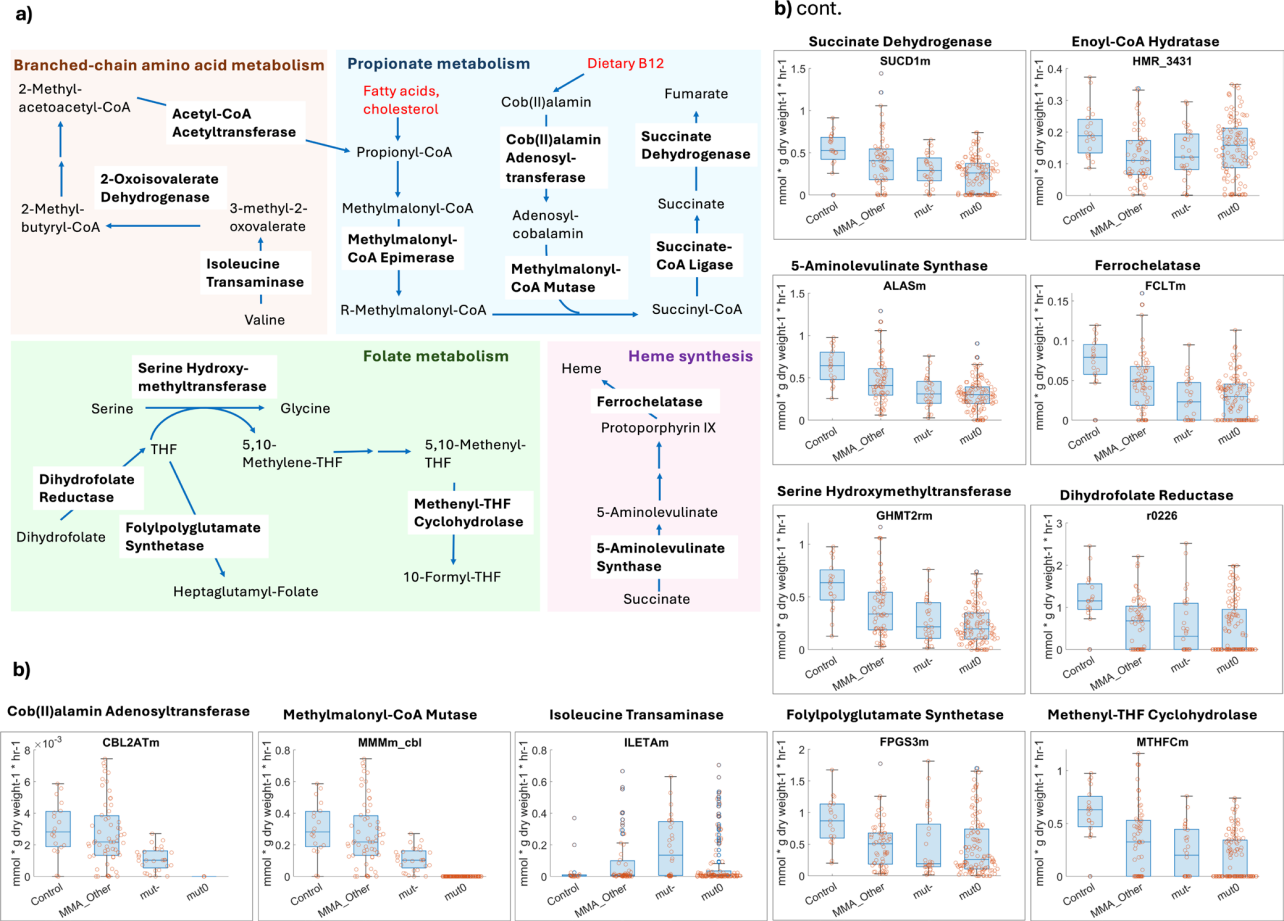
Overview of altered metabolic fluxes in subtypes of MMA patients compared with controls. (a) Schematic overview of the main pathways that were found to be altered in flux in MMA patients. (b) Metabolic fluxes predicted for patients and controls for reactions shown in (a). Reactions are displayed by Virtual Metabolic Human reaction ID [20] or reaction ID newly assigned in this study (Table S1). MMA_other = other types of methylmalonic aciduria; MUT^−^ = mut^−^ cases, MUT0 = mut^0^ cases.

Reactions most affected between MMA and controls were further inspected (Figure 3). Generally, metabolic fluxes in mut^0^, the situation in which the methylmalonyl‐CoA mutase enzyme is entirely deficient, were most severely impacted compared with controls. As expected, propionate metabolism, namely, the conversion of methylmalonyl‐CoA into succinyl‐CoA via methylmalonyl‐CoA epimerase and methylmalonyl‐CoA mutase, was completely blocked in mut^0^, reduced in mut^−^, and, surprisingly, also slightly reduced in other causes of MMA (Figure 3a,b). The conversion of cob(II)alamin into adenosylcobalamin, the cofactor for methylmalonyl‐CoA mutase, was similarly reduced in flux (Figure 3b). Because of the reduction in succinyl‐CoA production, the succinate‐CoA ligase and succinate dehydrogenase reactions were also impaired in flux, resulting in reduced succinate and fumarate availability (Figure 3a,b). Consistently, in metabolomic analyses of mut‐type MMA fibroblasts, markedly reduced levels of TCA cycle intermediates, including succinate and fumarate, had been found [7]. As already seen for the in silico gene knockouts (Figure 2), the reduced succinate production potential also resulted in a clear reduction in heme biosynthesis capability in all MMA groups, including non‐mut‐type MMA (Figure 3a,b). Notably, a variety of hematological symptoms are found in individuals affected by MMA, albeit not restricted to heme‐carrying erythrocytes [28]. Another pathway that was clearly impaired in flux was one‐carbon metabolism. Serine hydroxymethyltransferase, which catalyzes the synthesis of glycine from serine (Figure 3b), and dihydrofolate reductase, which synthesizes tetrahydrofolate from dihydrofolate, were reduced in flux in MMA (Figure 3b). Folylpolyglutamate synthase, a key regulator of one‐carbon metabolism [29], and methenyl‐tetrahydrofolate cyclohydrolase, which synthesizes 10‐formyltetrahydrofolate, were similarly drastically reduced in flux (Figure 3a,b). Notably, impaired folate metabolism flux was seen in all forms of MMA, including non‐mut‐type MMA (Figure 3b). Due to the key role of folate metabolism for the synthesis of methyl donors [30], this reduction in biosynthetic capability may cause impairment in the epigenetic machinery.

In contrast, an increase in the degradation of the branched‐chain amino acids valine and isoleucine into propionyl‐CoA compared with controls was seen specifically in mut^−^ (Figure 3a,b, Table S7). Isoleucine is a major source of propionyl‐CoA in fibroblasts [31] and increased flux through this pathway may suggest dysregulation of propionyl‐CoA flux. Finally, in non‐mut MMA samples, the most affected subsystem was fatty acid oxidation and synthesis (e.g., enoyl‐CoA dehydratase), which was mostly decreased in flux (Figure 3b, Table 2).

### 3.6 Associations Between Metabolic Fluxes and Clinical Presentation in MMA Patients

Individuals affected by MMA frequently present with severe symptoms including developmental delay, seizures, and metabolic decompensation [32]. However, there is large interindividual variation in the presence and severity of symptoms. For example, in a recent meta‐analysis, around 40% of individuals showed developmental delay, 21% displayed hypotonia, and 26% showed acute metabolic decompensation, while anemia was less frequent [4]. We hypothesized that differences in metabolic flux capabilities may contribute to this variability in presentation. To this end, we aimed to identify reaction fluxes associated with the presence or absence of symptoms. We retrieved clinical presentations and treatment interventions that had been reported for each individual [7] (Table S10). It should be noted that the absence of a symptom may also mean it was not reported. We then performed a Wilcoxon rank test for all reactions with minimal or maximal flux for all clinical features in individuals affected by MMA (see Section 2).

**FIGURE 4:**
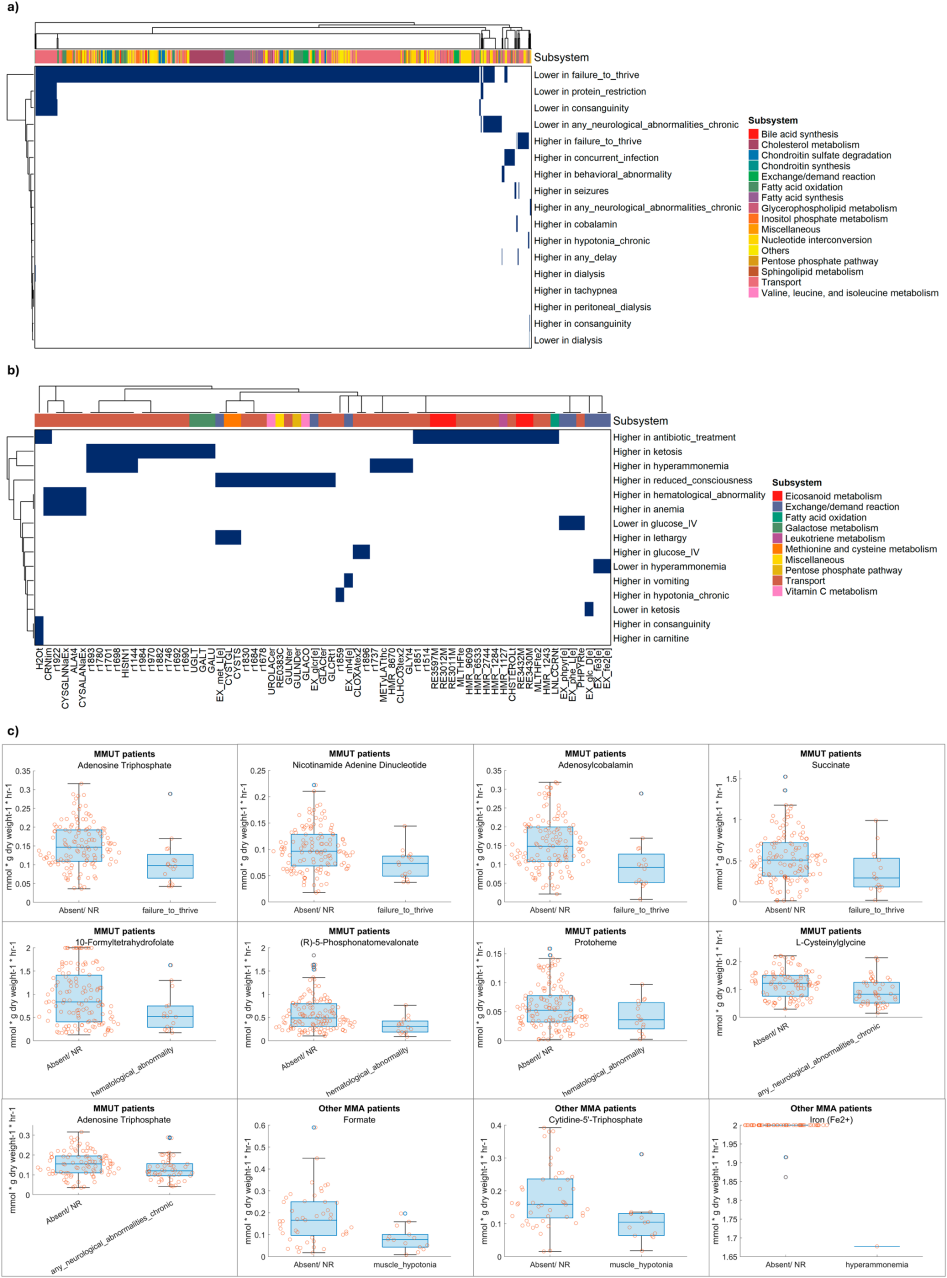
Flux differences associated with the presence of clinical symptoms in subtypes of MMA patients. Reactions that were significantly altered in flux in (a) mut‐type MMA, (b) non‐mut MMA displaying symptoms or undergoing treatment compared with patients where that was not the case or not reported. Columns contain reactions annotated by assigned subsystem. Rows contain symptoms and treatments. In (b), reactions are displayed by VMH reaction ID [20] or reaction ID newly assigned in this study (Table S1). (c) Metabolite production potential that differed between patients displaying symptoms or undergoing treatment compared with patients where that was not the case or not reported. NR = not reported. More details are shown in Tables S11–S14.

Reactions differing in flux between the presence and absence of symptoms were determined for mut‐type (both mut^0^ and mut^−^) MMA and other causes of MMA separately (Figure 4a,b). In total, 1380 reactions in mut‐type and 67 reactions in other causes were predicted to have either increased or decreased flux in those individuals with reported symptoms or treatments (Figure 4a, Tables S11 and S12). The greatest impact of symptoms in individuals affected by mut‐type MMA was seen in patients with failure to thrive, where 1277 reactions across a variety of metabolic subsystems, including transport, nucleotide interconversion, and fatty acid oxidation and synthesis, were reduced in flux, while 32 reactions were upregulated (Figure 4a, Table S11). This suggests a systemic dysregulation of metabolism in fibroblasts from patients with failure to thrive, consistent with the severity of the symptom. Mut‐type MMA patients treated through protein restriction were predicted to have lower flux for a total of 66 reactions, including arginine and proline metabolism (Figure 4a, Table S11), consistent with reduced amino acid availability due to the treatment. In non‐mut MMA, 19 reactions were upregulated in individuals that had received antibiotic treatment (Figure 4b, Table S12). This included reactions in eicosanoid and leukotriene metabolism, suggesting an association with increased inflammation (Figure 4b). In individuals with ketosis, galactose metabolism and transport were upregulated. Individuals with reduced consciousness were predicted to have increased flux in reactions related to methionine and cysteine metabolism and vitamin C metabolism, while those with hyperammonemia showed reduced iron uptake flux (VMH reaction IDs EX_fe2_e, EX_fe3_e) (Figure 4b). Taken together, both symptom presentations and interventions were associated with altered metabolic fluxes, which were specific to the underlying cause of MMA.

To confirm that the observed flux changes affected the bioavailability of metabolites in silico, we predicted the theoretical potential of each sample‐specific model to synthesize all nonunique internal metabolites (see Section 2). In total, 1136 nonunique metabolites in individuals affected by mut‐type MMA and 150 nonunique metabolites in other causes were predicted to have significantly different symptom‐specific flux changes (Figures S2 and S3, Tables S13 and S14). For instance, in mut‐type individuals with failure to thrive, 1051 metabolites had reduced biosynthesis potential, such as ATP, NAD, adenosylcobalamin, and succinate (Figure 4c, Figure S2, Table S13), suggesting impaired energy metabolism, cobalamin cofactor availability, and citric acid cycle flux. In mut‐type individuals with hematological abnormalities, 577 nonunique metabolites were predicted to have impaired biosynthesis potential (Figure S2, Table S13), including, for instance, the one‐carbon metabolite 10‐formyltetrahydrofolate and 5‐phosphomevalonate, a precursor of cholesterol biosynthesis (Figure 4c). Consistently, a reduction in heme biosynthesis potential was also seen in patients with hematological abnormality (Figure 4c). In patients with neurological abnormalities, 530 nonunique metabolites were reduced in biosynthesis potential (Figure S2, Table S13). This included, for instance, l‐cysteinylglycine and ATP (Figure 4c). In non‐MUT MMA, 129 nonunique metabolites were decreased in biosynthesis potential in individuals with muscle hypotonia (Figure S3), for instance, formate and cytidine triphosphate (Figure 4c). Moreover, in hyperammonemia, iron availability was reduced (Figure 4c, Table S14), consistent with reduced iron uptake flux (Figure 4b). Taken together, consistent with metabolic fluxes, the metabolite production potential across a variety of pathways was altered in individuals presenting with symptoms and specific to MMA subtypes.

## 4 Discussion

In this work, we systematically predicted perturbed metabolic fluxes associated with IECMs using a constraint‐based modeling approach. We found that 11 known IECMs could be grouped into three clusters based on reduced fluxes, which roughly corresponded to clusters that had been observed in a previous meta‐analysis grouping of IECM patients by symptoms [4]. Next, we focused our attention on the MMA subtype of IECMs. We found that reaction fluxes were clearly perturbed in patients with complete or partial deficiency in methylmalonyl‐CoA mutase, and to a lesser extent in other types of MMA. Finally, we found that differences in fluxes between absence and presence of symptoms were specific to mut‐type MMA and other types of MMA. Taken together, our modeling framework allowed for an in‐depth look at metabolic pathways that are altered in IECMs as well as at individual‐specific differences.

Our simulations indicate that fibroblasts from individuals with mut‐type MMA demonstrated impaired flux through multiple pathways. Indeed, our findings of reduced succinate availability, impaired heme biosynthesis, and inhibited conversion of succinate into fumarate are consistent with the reduction in TCA cycle enzymes and intermediates found previously in proteomic and metabolomic analyses of these cells [7]. Due to the key role of the TCA cycle in the regulation of central metabolism, these impairments may partially explain the global metabolic perturbations seen in individuals affected by MMA, such as metabolic acidosis and hyperammonemia [32]. We also found folate metabolism flux to be reduced in MMA. The involvement of mitochondrial folylglutamate synthase and serine hydroxymethyltransferase indicates a mitochondrial specific perturbation, consistent with the location of the methylmalonyl‐CoA mutase enzyme. Due to the importance of folates for the synthesis of methyl donors, this perturbed folate metabolism may also affect DNA methylation [1].

Decreased activity in methylmalonyl‐CoA mutase is not restricted to gene defects in the mitochondrial cluster of IECMs, which includes the non‐mut, mut^−^, and mut^0^ subtypes considered in our study [4]. Pathogenic variants in any genes involved in the intracellular transport of cobalamin produce a decreased cellular availability of cobalamin and a subsequent combined deficit in both methylmalonyl‐CoA mutase and methionine synthase activities [4]. The most frequent type of IECM, cblC, is due to mutations in the *MMCHC* gene [4]. In contrast, the cblG type, which is associated with mutations in the *MTR* gene, decreases only the activity of methionine synthase [1]. Our estimates of fluxes of propionate, succinate, and fumarate in Clusters (III) and (IV) are concordant with the increased propionate and decreased succinate and fumarate concentrations that we observed previously in a multi‐omic analysis of fibroblasts from patients with cblC but not in those with cblG [5]. The decreased fluxes in fatty acid oxidation and synthesis, succinyl CoA:3‐oxoacid CoA transferase, and carnitine palmitoyltransferase were also consistent with the altered expression of genes involved in fatty acid metabolism and the decreased expression of *OXCT1* and *CPT2* that we observed in fibroblasts from cblC patients [5].

Transcobalamin (TCN2) is the protein involved in the CD320 receptor‐mediated cellular uptake of cobalamin [1]. TCN2 deficiency reduces the cellular availability of cobalamin and the activity of methylmalonyl‐CoA mutase. We previously showed that the *TCN2* c.776*GG* genotype is twofold more frequent in Afro‐African individuals with severe malaria than in other Afro‐Africans exposed to malaria, with a Hardy–Weinberg disequilibrium of *TCN2* genotype distribution recorded only in patients with severe malaria [33]. Our predictions here that heme biosynthesis is reduced in IECMs are consistent with our former hypothesis that the *TCN2* c.776*GG* genotype produces a higher risk of severe malaria related to cobalamin‐related decreased heme synthesis [33]. We did not identify any changes that could clearly be related to the reduced production of white blood cells or platelets reported in IECMs. However, this is likely not surprising, as the “toxic metabolite” effects could happen directly at the protein level and not be accounted for in our model. For example, one of the expected tissue‐specific effects of metabolites is the generation of posttranslational modifications, as recently reported in fibroblasts of IECMs [5].

The clinical presentation of MMA is very variable, and more insight is needed into the underlying causes for the variability demonstrated in previous meta‐analyses and multi‐omics studies [5, 7]. Our metabolic modeling provides testable hypotheses for pathways altered in specific subsets of patients that may lead to symptoms. For instance, we found that energy metabolites, including ATP and NAD, were predicted to be reduced in individuals with mut‐type MMA who demonstrated failure to thrive. Based on our simulations, a chronic reduction in energy availability might play a role in delayed and impaired development in these patients. This prediction could be validated in future studies.

Our study has limitations. First, we relied on fibroblast RNA sequencing data. Fibroblasts derived from skin biopsies are the most common in vitro model in IEM due to their accessibility and ease of preparation, and the conservation of most metabolic pathways in this cell line [34, 35]. The representation of more specialized functions occurring elsewhere in the body would require different patient‐specific cell lines, which are, however, not readily accessible [34]. In particular, measurements and constraint‐based analyses of cell types linked to disease‐specific pathophysiology, such as kidney cells in the case of MMA‐related renal impairment, could lead to a deeper understanding of disease mechanisms. Moreover, our study is subject to the known limitations of constraint‐based modeling. It operates under the steady‐state assumption, where metabolites are neither accumulating nor depleting, which results in the prediction of fluxes rather than concentrations [19]. This assumption has the advantage that kinetic parameters are not required, but the disadvantage that enzyme kinetics are not accounted for [11]. Additionally, due to the inherent flexibility in the metabolic network, simulation results represent a space of feasible solutions rather than a single possible flux distribution [11]. The accuracy of predicted flux solutions also depends on the quality of the underlying genome‐scale reconstruction. While Recon3D has undergone over a decade of manual curation [12], reactions and genes may be missing due to current knowledge gaps. We also acknowledge that we did not correct for patient age or sex in our analyses, which may be confounding factors. Finally, symptoms considered absent in our analysis may have been present in the patients but not reported in the documentation [7].

In future applications, systems biology could guide the developments of dietary or therapeutic interventions for IEMs [14]. Current treatment options for MMA include hydroxocobalamin supplementation, to which, unfortunately, only a subset of patients respond [6]. Other treatment options include a specialized diet low in precursor amino acids, carnitine supplementation, and intermittent antibiotics [21], but the treatment of MMA remains challenging. The genome‐scale modeling approach presented in this study could be used to predict personalized treatments for MMA [14]. Such predictions could be validated in organoids or whole animal models. Alternatively, the in silico whole‐body model, Harvey, which can be contextualized with dietary information and physiological parameters of patients [36], could be used to predict metabolic fluxes in organs other than skin, which would more accurately reflect symptoms in MMA. Harvey previously enabled the prediction of biofluid‐specific biomarkers for IEMs with good accuracy [36]. In future studies, Harvey could additionally be parameterized with omics data (e.g., cell‐type specific transcriptomics) and propose targeted dietary or therapeutic interventions [14].

## 5 Conclusion

In conclusion, we have systematically explored metabolic reactions and pathways perturbed in individuals with IECMs. We found that integrating sample‐specific omics data further informed our simulations and revealed the individual variability in individuals with different types of MMA and symptom presentations. To our knowledge, this is the first constraint‐based modeling study applying personalized modeling to stratify patients with rare diseases. In future studies, the presented personalized modeling pipeline could be applied for the prediction of biomarkers that distinguish between different IECMs [14]. Moreover, using, for example, the whole‐body human model, targeted dietary and therapeutic interventions for individuals with IECMs could be predicted [14], which ultimately could improve the management of these severe, difficult to treat diseases. Hence, in future efforts, personalized metabolic modeling may pave the way to an omics‐based, patient‐specific precision medicine strategy [10] to diagnose and treat rare diseases.

## Author Contributions

A.H., J.‐L.G., R.‐M.G.‐R., and D.S.F. designed the study. A.H. performed simulations. A.H. and H.A. analyzed data. V.R.T.Z. and D.S.F. provided the fibroblast data and patient metadata. A.H. drafted the manuscript. A.H., D.S.F., J.‐L.G., and V.R.T.Z. revised the manuscript. A.H., J.‐L.G., and R.‐M.G.‐R. supervised the project. All authors reviewed and approved the final version of the manuscript.

## Ethics Statement

No ethics approval was required as all input data had been published previously.

## Consent

No patient consent was required as all input data had been published previously.

## Conflicts of Interest

The authors declare no conflicts of interest.

## Supporting information


**Figure S1:** Overlap between reactions reduced in flux in the Recon3D MMUT complete KO scenario (Table S2); and between reactions that were significantly different between mut^0^ and control personalized fibroblast models (Table S5).
**Figure S2:** Metabolites that were significantly altered in production capacity in mut‐type MMA displaying symptoms or undergoing treatment compared with patients where that was not the case or not reported. Columns contain nonunique metabolites. Rows contain symptoms and treatments.
**Figure S3:** Metabolites that were significantly altered in production capacity in other MMA cases displaying symptoms or undergoing treatment compared with patients where that was not the case or not reported. Columns contain nonunique metabolites. Rows contain symptoms and treatments.

**Table S1:** Reactions, metabolites, and genes that were added to Recon3D or corrected.
**Table S2:** Constraints representing the simulated Dulbecco's modified Eagle's medium (DMEM) medium.
**Table S3:** Reactions that were reduced in flux by at least 10% in at least one simulated IECM.
**Table S4:** Features of each personalized model and sample.
**Table S5:** Minimal and maximal fluxes computed for the 221 personalized models generated in this study.
**Table S6:** Statistical analysis for reaction fluxes between mut^0^‐type MMA patients and controls.
**Table S7:** Statistical analysis for reaction fluxes between mut^−^‐type MMA patients and controls.
**Table S8:** Statistical analysis for reaction fluxes between mut^0^‐type and mut^−^‐type MMA patients.
**Table S9:** Statistical analysis for reaction fluxes of non‐MMUT MMA patients and controls.
**Table S10:** Description of symptoms that had been reported for patients.
**Table S11:** Reactions that were predicted to be significantly lower or higher in flux in the presence of symptoms in MMUT patients.
**Table S12:** Reactions that were predicted to be significantly lower or higher in flux in the presence of symptoms in non‐MMUT MMA patients.
**Table S13:** Metabolites that were predicted to be significantly lower or higher in biosynthesis flux in the presence of symptoms in MMUT patients.
**Table S14**: Metabolites that were predicted to be significantly lower or higher in biosynthesis flux in the presence of symptoms in non‐MMUT MMA patients.

## Data Availability

Scripts and input data that enable reproducing the simulations and analyses are available at https://github.com/almut‐heinken/ngereSysBio, reference number 3ab13a7.
