# Supplementary material for: Personalized Genome‐Scale Modeling Reveals Metabolic Perturbations in Fibroblasts of Methylmalonic Aciduria Patients
Source: J Inherit Metab Dis. 2025 Aug 11;48(5):e70077. doi: 10.1002/jimd.70077 (PMC12340171; doi:10.1002/jimd.70077)
Supplement: Supplementary file 1 — Figure S1: Overlap between reactions reduced in flux in the Recon3D MMUT complete KO scenario (Table S2); and between reactions that were significantly different between mut0 and control personalized fibroblast models (Table S5). Figure S2: Metabolites that were significantly altered in production capacity in mut‐type MMA displaying symptoms or undergoing treatment compared with patients where that was not the case or not reported. Columns contain nonunique metabolites. Rows contain symptoms and treatments. Figure S3: Metabolites that were significantly altered in production capacity in other MMA cases displaying symptoms or undergoing treatment compared with patients where that was not the case or not reported. Columns contain nonunique metabolites. Rows contain symptoms and treatments. [file JIMD-48-0-s001.docx]

**Supplemental figures for: Personalized genome-scale modeling reveals metabolic perturbations in fibroblasts of methylmalonic aciduria patients**

Almut Heinken^1$^, Hussein Awada^1^, Vito R.T. Zanotelli^2^, D. Sean Froese^2^, Rosa-Maria Guéant-Rodriguez^1,3^, and Jean-Louis Guéant^1,3,4^

^1^ Faculty of Medicine of Nancy INSERM U1256, Nutrition, Genetics, and Environmental Risk Exposure (NGERE), University of Lorraine, Vandœuvre-les-Nancy, France

^2^Division of Metabolism and Children's Research Center, University Children's Hospital Zürich, University of Zürich, Zürich, Switzerland

^3^University Hospital Nancy, Reference Medical Biology Laboratory (LBMR), Biochemistry Molecular Biology Nutrition Laboratory, Vandœuvre-les-Nancy, France

^4^Institute of Medical Research (Pôle BMS) - University of Lorraine, Vandœuvre-les-Nancy, France


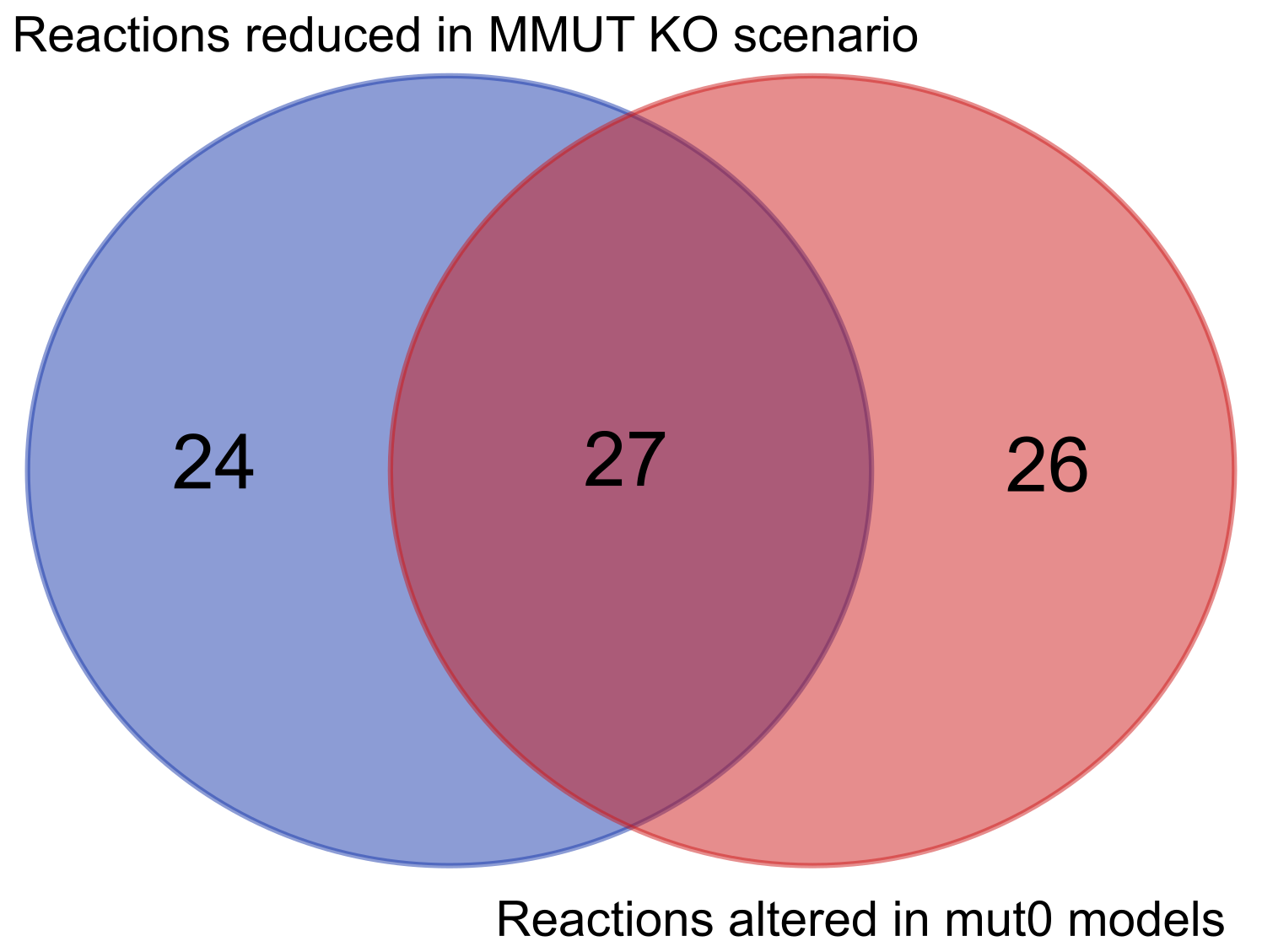


**Figure S1**: Overlap between reactions reduced in flux in the Recon3D MMUT complete KO scenario (Table S2), and between reactions that were significantly different between mut^0^ and control personalized fibroblast models (Table S5).


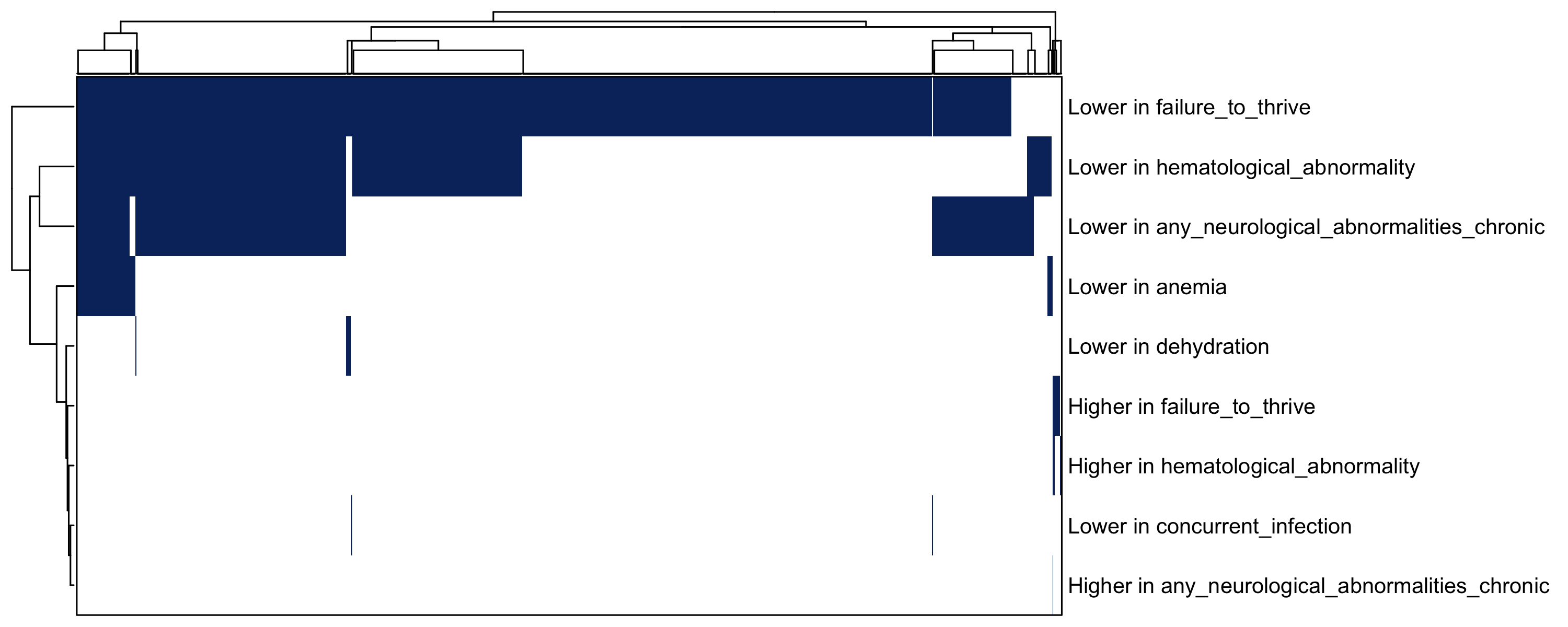


**Figure S2**: Metabolites that were significantly altered in production capacity in mut-type MMA displaying symptoms or undergoing treatment compared with patients where that was not the case or not reported. Columns contain non-unique metabolites. Rows contain symptoms and treatments.


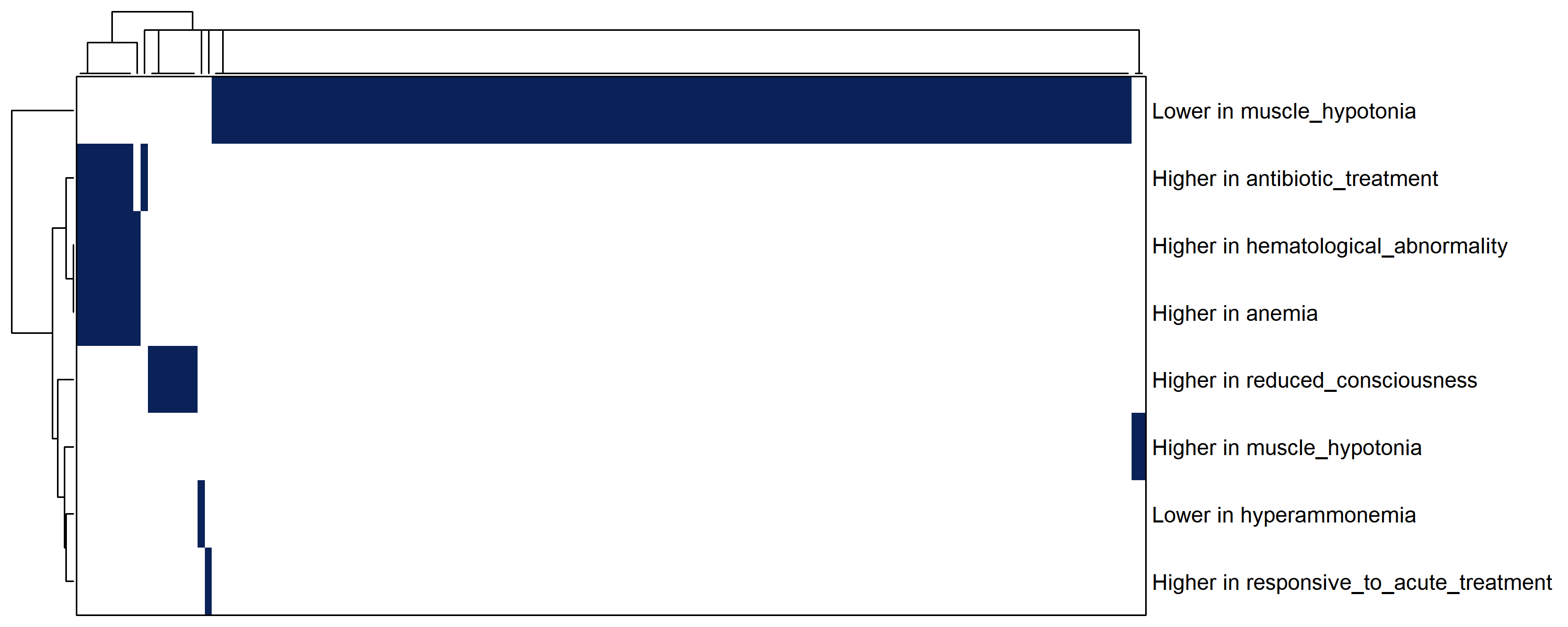


**Figure S3**: Metabolites that were significantly altered in production capacity in other MMA cases displaying symptoms or undergoing treatment compared with patients where that was not the case or not reported. Columns contain non-unique metabolites. Rows contain symptoms and treatments.
